# Supplementary figures and images for: 14-3-3 mitigates alpha-synuclein aggregation and toxicity in the in vivo preformed fibril model
Source: Acta Neuropathol Commun. 2021 Jan 7;9:13. doi: 10.1186/s40478-020-01110-5 (PMC7792107; doi:10.1186/s40478-020-01110-5)

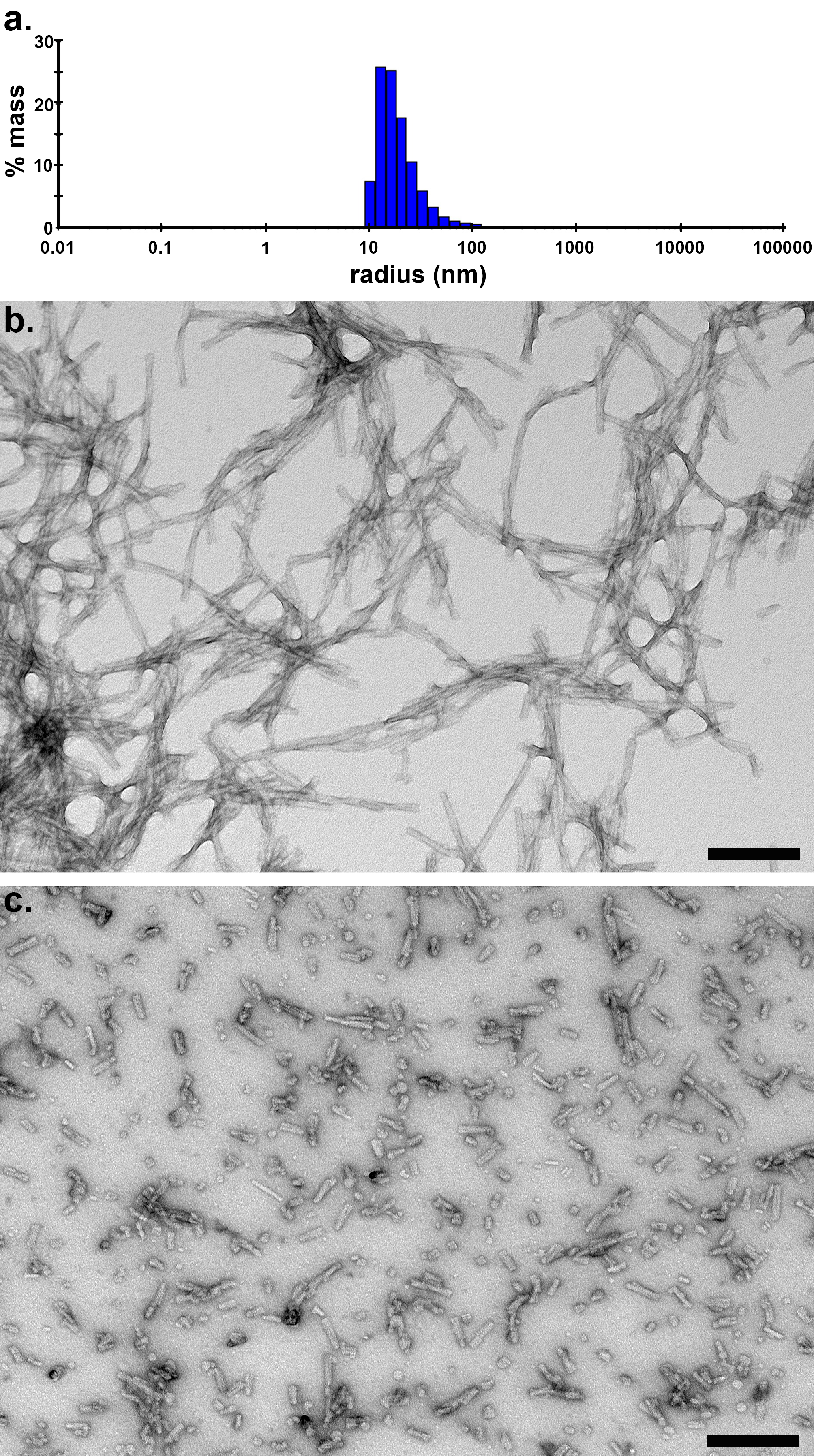

Supplement: Supplementary file 1 — Additional file 1: Figure S1 Verification of sonicated fibril radius by dynamic light scattering (DLS). (a) Representative DLS graph of average sonicated fibril radius shown for WT and 14-3-3θ transgenic mice injected for the 6 mpi time point. After sonication, PFFs were confirmed by Nanodrop for concentration and analyzed by DLS to ensure a mean radius of 20-50 nm for sonicated fibrils. PFFs were reassessed by DLS at the beginning of each day of injections. (b) Representative TEM of αsyn fibrils before sonication. Scale bar = 200 nm. (c) Representative TEM of αsyn fibrils after sonication. Scale bar = 200 nm. [file 40478_2020_1110_MOESM1_ESM.tif]

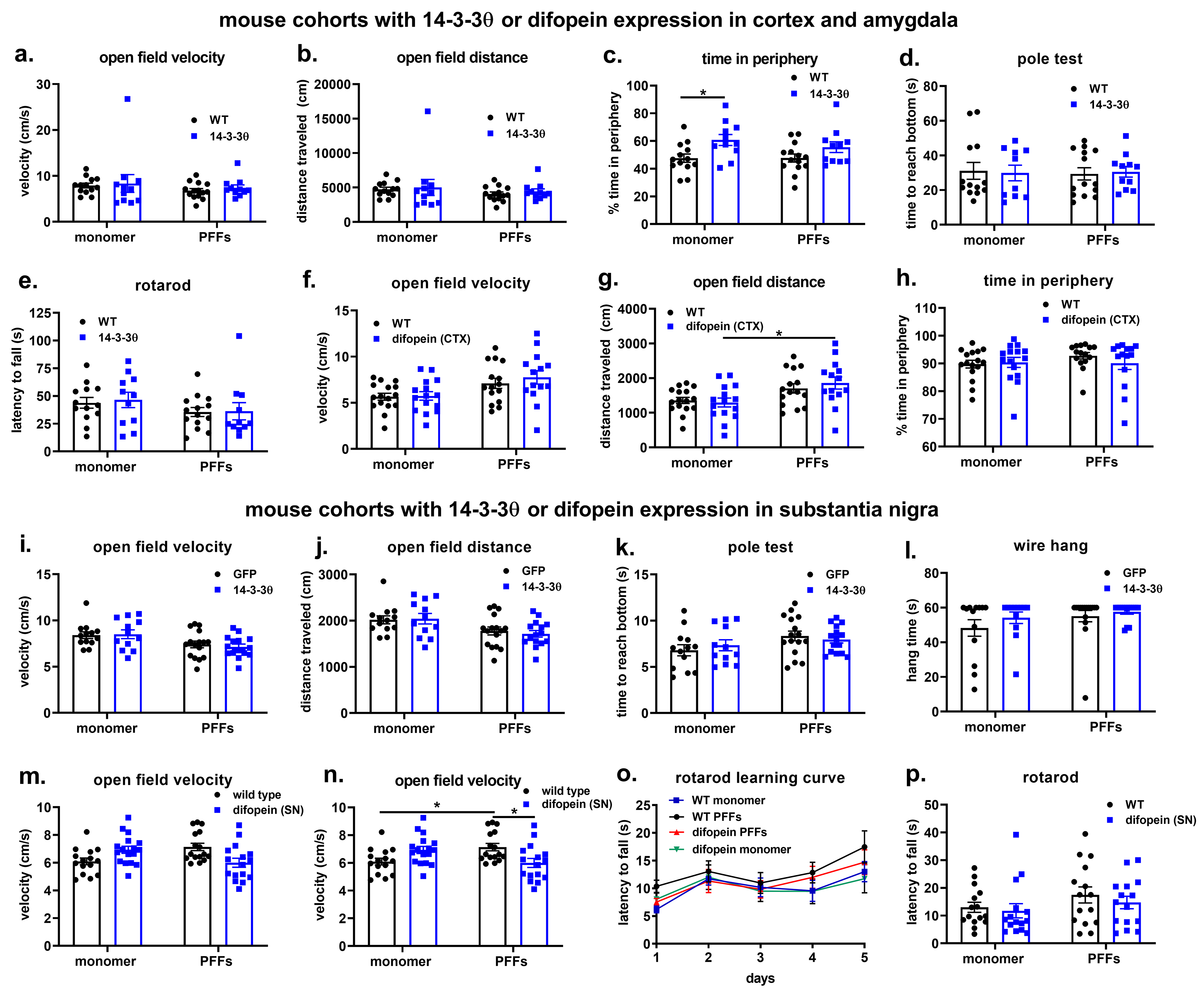

Supplement: Supplementary file 2 — Additional file 2: Figure S2 Motor behaviors are not affected by PFF treatment nor by 14-3-3 manipulation in the cortex, amygdala, or nigra. (a–c) Quantification of average velocity (a), distance traveled (b), and percent time in the periphery (c) in the open field test for WT and 14-3-3θ mice injected with αsyn monomer or PFFs at 6 mpi. n = 11–14 per group, *p < 0.05 (Tukey’s multiple comparison test) (Tukey’s multiple comparison test). Error bars represent SEM. (d) Quantification of time to reach the bottom in the pole test for WT and 14-3-3θ mice injected with αsyn monomer or PFFs at 6 mpi. n = 11–14 per group, n.s. (Tukey’s multiple comparison test). Error bars represent SEM. (e) Quantification of latency to fall in the accelerating rotarod test on the second assessment day after 3 days of training for WT and 14-3-3θ mice injected with αsyn monomer or PFFs at 6 mpi. n = 11–14 per group, n.s. (Tukey’s multiple comparison test). Error bars represent SEM. (f, h) Quantification of average velocity (f), distance traveled (g), and time in periphery (h) in the open field test for WT and difopein (“cortical” line 138) mice injected with αsyn monomer or PFFs at 6 mpi. n = 14–16 per group, *p < 0.05 (Tukey’s multiple comparison test). Error bars represent SEM. (i–l) Quantification of average velocity (i) and distance traveled (j) in the open field test, time to reach the bottom in the pole test (k), and latency to fall on the wire hang test (l) in AAV-GFP and AAV-14-3-3θ/GFP mice injected with αsyn monomer or PFFs at 6 mpi. n = 12–16 per group. n.s. (Tukey’s multiple comparison test). Error bars represent SEM. (m–p) Quantification of average velocity (m) and distance traveled (n) in the open field test and latency to fall in the accelerating rotarod test (o, p) in WT and difopein (“nigral” line 166) mice injected with αsyn monomer or PFFs at 6 mpi. n = 15–17 per group. *p < 0.05 (Tukey’s multiple comparison test). Error bars represent SEM. [file 40478_2020_1110_MOESM2_ESM.tif]

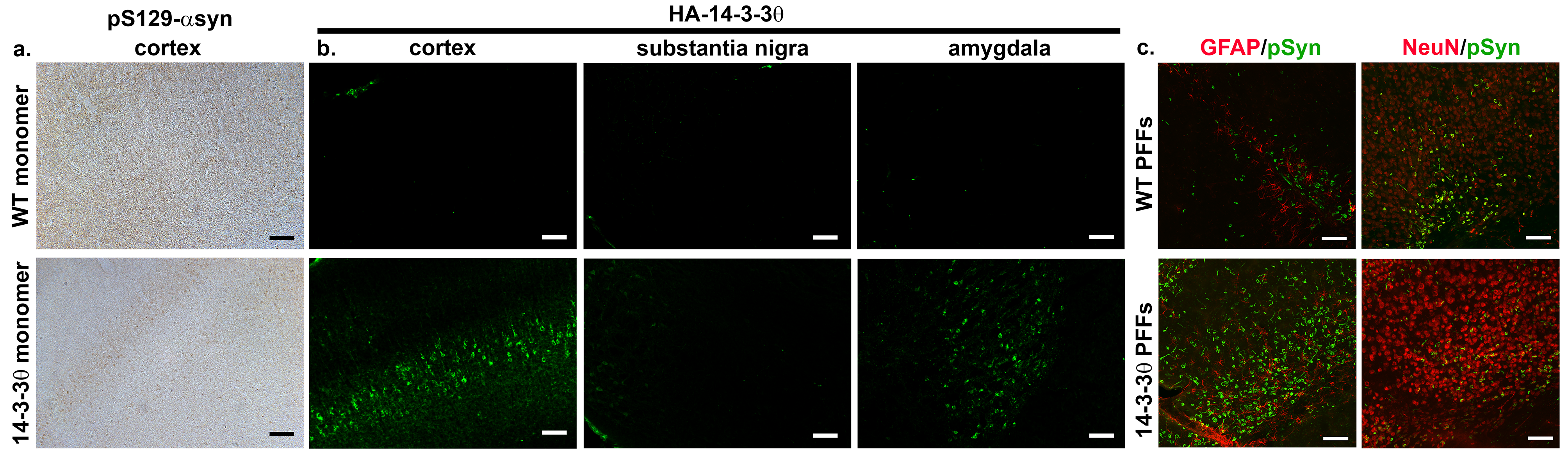

Supplement: Supplementary file 3 — Additional file 3: Figure S3 αSyn aggregation occurs primarily in neurons at 6 months post injection. (a) Representative images of pS129-αsyn immunostaining in the cortex at 6 mpi in WT and 14-3-3θ mice injected with αsyn monomer into the striatum. Scale bar = 100 μm. (b) Representative images of HA immunostaining in the cortex, SN, and amygdala demonstrates that HA-tagged 14-3-3θ is expressed in cortical and amygdala regions, but not in the SN. Scale bar = 100 μm for cortex and SN; 50 μm for amygdala. (c) pS129-αsyn inclusions associate primarily with NeuN instead of GFAP in PFF-injected WT and 14-3-3θ transgenic mice at 6 mpi. Scale bar = 50 μm. [file 40478_2020_1110_MOESM3_ESM.tif]

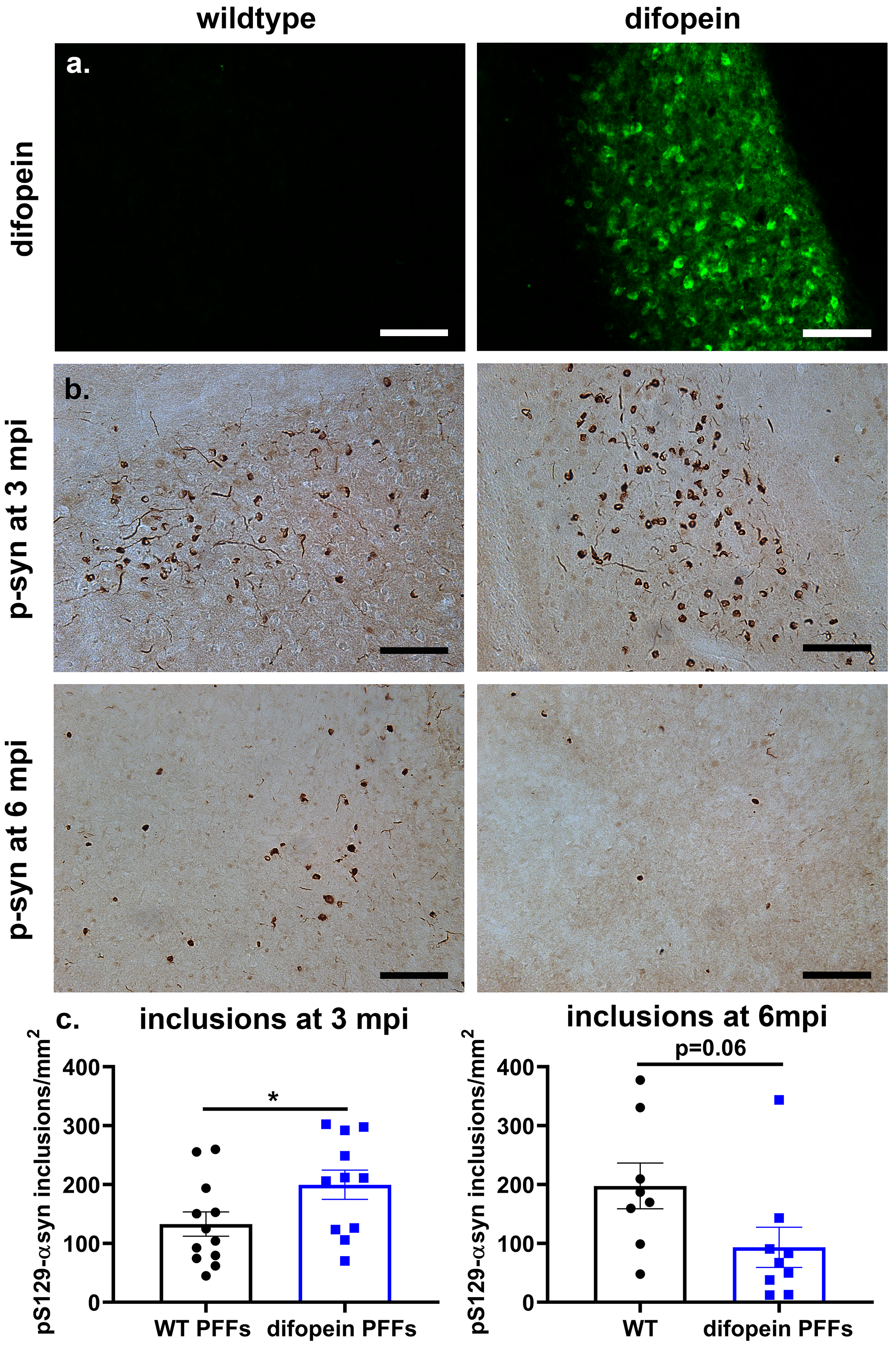

Supplement: Supplementary file 4 — Additional file 4: Figure S4 αSyn aggregation is accelerated in the amygdala in mice expressing difopein in the amygdala. (a) Representative images of eYFP-difopein immunostaining in the amygdala of WT and difopein (“cortical” line 138) mice at 3 mpi. GFP-difopein expression is found only in difopein mice. Scale bar = 100 μm. (b) Representative images of pS129-αsyn immunostaining in the cortex of WT and difopein mice at 3 and 6 mpi. Scale bar = 100 μm. (c) Difopein expression increases inclusion counts in PFF-injected mice at 3 mpi. Quantification of pS129-αsyn positive inclusions at 3 mpi and 6 mpi in the amygdala of PFF-injected WT and difopein mice. n = 11–12 per group at 3 mpi. n = 8–9 at 6 mpi. *p < 0.05 (Student’s t-test). Error bars represent SEM. [file 40478_2020_1110_MOESM4_ESM.tif]

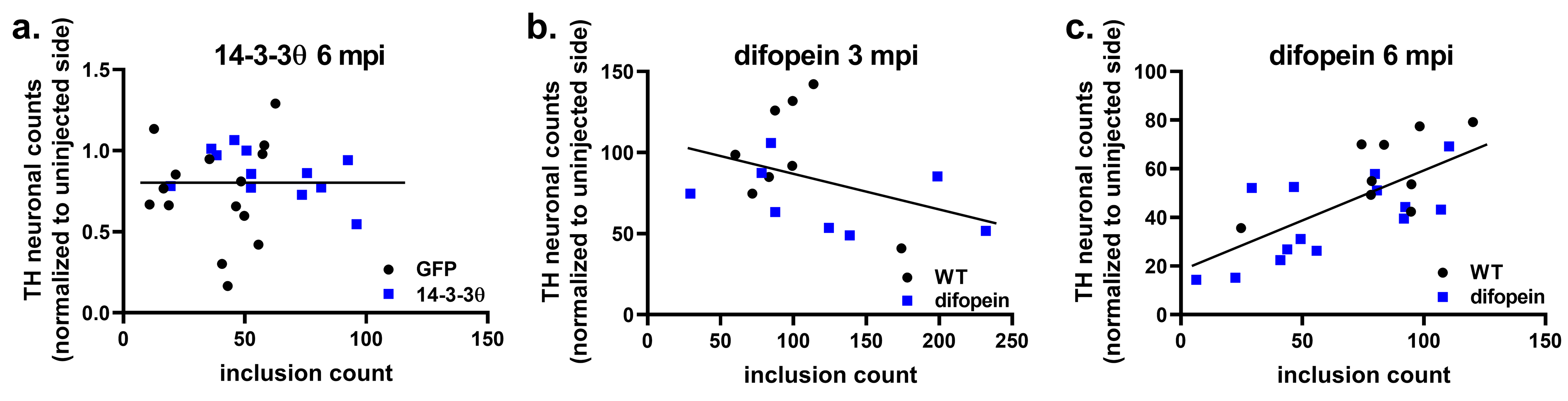

Supplement: Supplementary file 5 — Additional file 5: Figure S5 pS129-αsyn positive inclusions do not consistently correlate with TH-positive neuronal cell counts. (a) pS129-αsyn positive inclusions and TH-positive cell counts in PFF-injected AAV-14-3-3θ/GFP and AAV-GFP mice at 6 mpi (Pearson r = −0.00369, p = 0.9854). (b) pS129-αsyn positive inclusions and TH-positive cell counts in PFF-injected difopein and WT mice at 3 mpi (Pearson r = −0.3814, p = 0.145). (c) pS129-αsyn positive inclusions and TH-positive cell counts in PFF-injected difopein and WT mice at 6 mpi (Pearson r = 0.7157, p = 0.0001). [file 40478_2020_1110_MOESM5_ESM.tif]

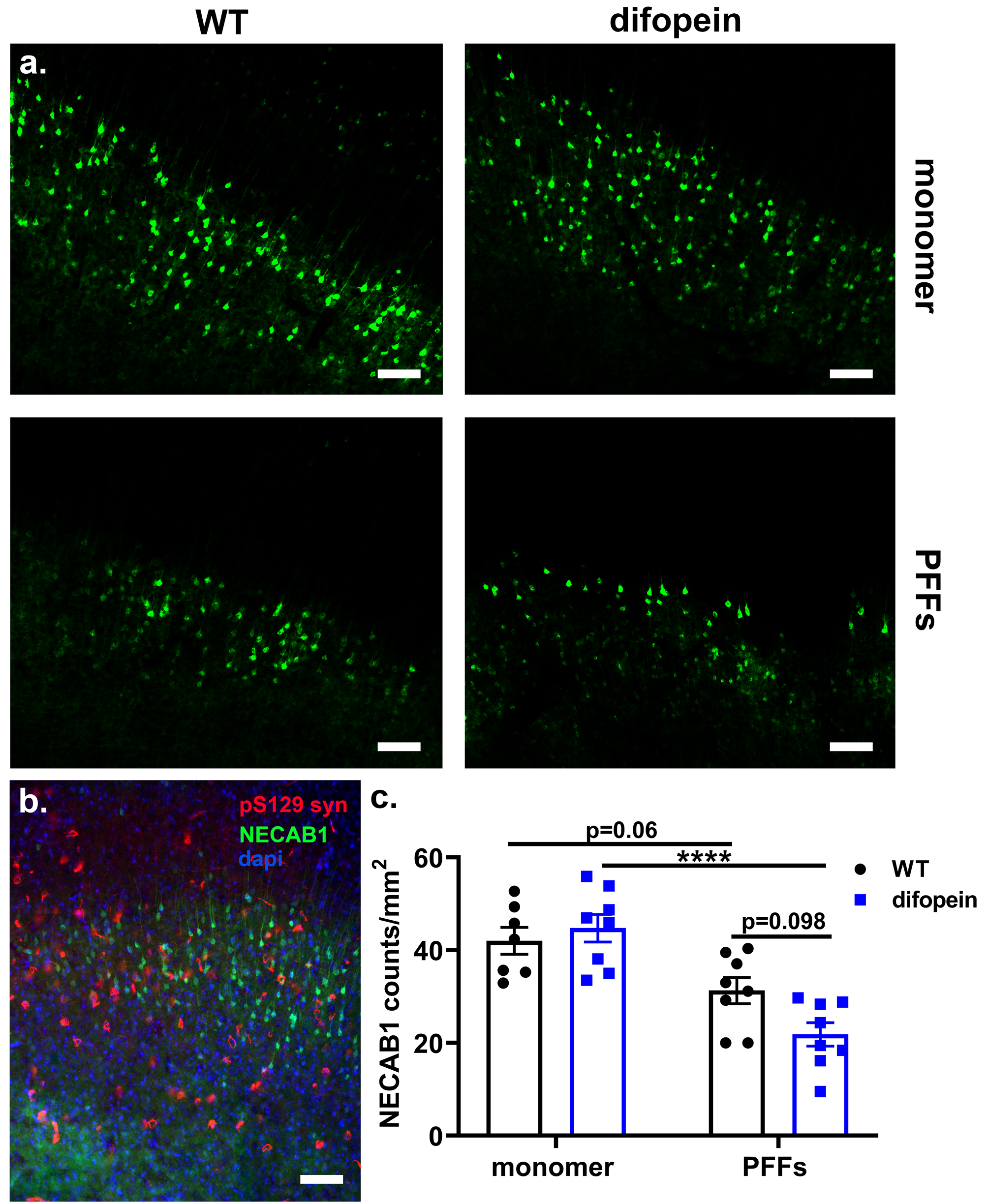

Supplement: Supplementary file 6 — Additional file 6: Figure S6 Difopein promotes the reduction of NECAB1-positive neurons in response to PFFs. (a) Representative images of NECAB1-positive immunostaining in the cortex of WT and difopein mice injected with αsyn monomers or PFFs at 6 mpi. Scale bar = 100 μm. (b) pS129-αsyn (red) immunostaining is concentrated in NECAB1-positive (green) IV and V layers of the sensorimotor cortex in a PFF-injected WT mouse. Scale bar = 50 μm. (c) PFF-injected difopein mice have decreased NECAB1 counts in comparison to monomer-injected difopein mice. Quantification of NECAB1-positive neurons in the cortex of WT and difopein mice injected with αsyn monomers or PFFs at 6 mpi. n = 7–8 per group. ****p < 0.0001 (Tukey’s multiple comparison test). Error bars represent SEM. [file 40478_2020_1110_MOESM6_ESM.tif]
